# Examining the Temporal and Spatial Models of China’s Circular Economy Based upon Detailed Data of E-Plastic Recycling

**DOI:** 10.3390/ijerph19052807

**Published:** 2022-02-28

**Authors:** Yu Qi, Ruying Gong, Xianlai Zeng, Junfeng Wang

**Affiliations:** 1 College of Environmental Science and Engineering, Nankai University, Tianjin 300350, China; qiyu@nankai.edu.cn; 2 Research Center for Resource, Energy and Environmental Policy, Nankai University, Tianjin 300350, China; 3 Department of Horticulture, Beijing Vocational College of Agriculture, Beijing 102442, China; gongruying@bvca.edu.cn; 4 State Key Joint Laboratory of Environment Simulation and Pollution Control, School of Environment, Tsinghua University, Beijing 100084, China

**Keywords:** electronic waste, plastic, circular economy, anthropogenic circularity, model

## Abstract

Examining the circular economy model is crucial to enable the scaling up of industry and anthropogenic circularity practice. Electrical and electronic waste plastic (e-plastic) has become the focus of urban mining and circular economy due to its rapid growth, valuable resource and potential risks. This article focuses on the recycling companies’ experience in China from 2012 to 2017. The average recycling rate was 33.3% and the recycling amount in 2017 was 558 kt. A two-dimensional coupling model of economic development and renewable resources is firstly constructed. Eventually, four typical resource-based regional models are summarized, namely for demonstration regional model, commissioned regional model, traditional model and potential regional model. It also puts forward differentiated suggestions in terms of maintaining demonstration, strengthening policies, promoting transformation, and tapping potential. At the same time, it is recommended to explore the construction of large-region resource-based recycling centers and big data centers in resource-based demonstration areas.

## 1. Introduction

Waste plastics is an emerging environmental issue of recent global concern. China has formulated policies such as bans and restrictions on plastics to promote the management of plastics. Electronic waste (e-waste) mainly includes various waste home appliances, communication equipment and products, and precision electronic instruments and meters discarded by enterprises and institutions. Waste home appliances are the main source. The plastic content in waste home appliances accounts for 10–20% of the total weight of plastics consumed in China throughout the year [1,2]. Some previous literatures indicated that the plastic content of waste household appliances in the United States accounts for roughly 30% of the total annual plastic consumption, the European Union about 40%, and Japan around 50%, all of which are significantly higher than that in China [3,4]. Among the discarded household appliances are TV sets, washing machines, air conditioners, refrigerators, and microcomputers, called “four machines and microcomputer” in Chinese. Recycling of plastics from e-waste (e-plastic) is of great significance for improving the efficiency of plastic resource utilization and reducing the cumulative environmental impact [5].

Technical processes of resource recycling of waste plastics from household appliances mainly include direct melting, thermal cracking, and energy recovery [6]. Direct melting refers to a method in which waste plastics are reheated and plasticized after pretreatment steps such as sorting, crushing, and drying, and then used. Thermal cracking refers to the method of heating waste plastics at high temperature under oxygen-free and low-oxygen conditions to break C-C bonds and C-H bonds, and re-polymerizing free radicals to generate smaller molecules [7]. Energy recovery refers to a method of incineration of waste plastics that are difficult to reuse to generate heat. Basically, the selection of the abovementioned technologies depends upon the quality of waste plastics. Direct melting is used for higher-quality waste plastics, thermal cracking for medium-quality waste plastics, and incineration for energy recovery for lower-quality waste plastics [8].

In terms of e-waste recycling policies, developed countries mainly adopt the extended producer responsibility (ERP) system, which extends the responsibility of producers to the full life cycle of products, especially recycling, resource utilization and detoxification disposal. The European Union has formulated the detailed laws and regulations on electronic waste governance. *The Wasted Electrical and Electronic Equipment Directive* (WEEE Directive) is a representative document. The United States has promulgated *the Resource Protection and Recycling Law*. Japan has promulgated *the Law on Promoting Effective Utilization of Resources*, which clarifies the minimum ratio of recycling of various electronic wastes. Through comparison, China can further learn from experience in terms of the accuracy of laws and regulations and the diversity of means. Over two decades, China has tightened up the laws and regulations related to e-waste. The first is the guiding policies in the basic laws, the second is the special management regulations and standards for waste electrical and electronic products, and the third is the technical specifications and management regulations for the recycling of waste plastics [9]. However, in practice, there are also serious problems such as insufficient policy implementation and lack of resource utilization standards for waste plastics from household appliances [10].

Currently, there are inadequate studies on the resource utilization of e-waste plastics. Most of the existing studies do not distinguish waste plastics from other waste, and mainly focus on the classification and sorting process, and physical separation [11,12]. There is a lack of systematic and in-depth analysis on e-plastic recycling, especially summarizing the typical patterns of regionally differentiated resource utilization so as to formulate regionally differentiated resource policies. Overall, regarding the resource utilization of e-plastics, it is necessary to examine two perspectives. One is to conduct studies from the perspective of improving the efficiency of resource utilization, and the other is derived from the perspective of reducing the cumulative impact of the environment. Either way, there is a need to first conduct research on the standardized recycling volume and typical patterns of resource utilization of e-plastics in China so as to understand the basis for benchmark.

## 2. Materials and Methods

### 2.1. Brief Analysis of Classification of Existing Estimation Methods

Currently, the idea of estimating the content of plastics from e-waste is mainly based upon the different collection methods of waste. There are two main estimation methods. The first is to directly measure the collected e-waste in its original mixed state without classification. For example, Stenvall (2013) uses infrared spectroscopy to measure the plastic composition of three different sources of e-waste batches [13]. The plastics used in the composition analysis are mainly based on random selection from the real waste streams from 14 samples out of three batches. The results show that the main components are high-impact polystyrene (HIPS, 42 wt%), acrylonitrile-butadiene-styrene copolymer (ABS, 38 wt%), and polypropylene (PP, 10 wt%). The disadvantage of this method is that there are considerable differences in the measurement even within one batch. The second is to firstly classify the collected e-waste, and then measure the specific weights of different types of plastics in each category. For example, Martinho (2012) divides e-waste into cooling equipment, small electronic and electrical waste, printers, copying equipment, central processing unit (CPU), cathode-ray tube (CRT) monitors, and CRT TV sets [14]. A total of about 3400 pieces of equipment have been measured. The results show that the main components are polystyrene (PS), ABS, polycarbonate (PC)/ABS, HIPS, and PP.

### 2.2. Construction of Estimation Method

This article estimates the content of waste plastics from the “four machines and microcomputer” collected in China. The estimation mainly includes two steps. First is to estimate the proportion of all plastics in the “four machines and microcomputer”, and the second is to estimate the proportion of the main types of waste plastics in the “four machines and microcomputer”. According to the actual situation of the existing estimation methods, this article separately estimates the “four machines” and “microcomputer”.

As for the “four machines”, in the first step, this article mainly refers to Oguchi’s estimation methods and results [15]. In the second step, this article mainly refers to Yang (2011)’s estimation methods and results [16]. Oguchi (2011) divided the electronic and electrical waste into 21 categories [15], and obtained data on the proportion of waste plastics in 9 categories according to relevant literature. The 12 remaining categories and a total of 62 kinds of scrap products were dismantled, and the waste plastics they contained were weighed to calculate the proportion of waste plastics. We use the estimation results of refrigerators, washing machines, air conditioners, CRT TVs, plasma TVs, and liquid crystal display (LCD) TVs. Yang (2011) dismantled the “four machines” collected in China and obtained the proportion of the main types of waste plastics [16]. The main types of waste plastics include five types, namely ABS, PS, PP, polyethylene (PE), and polyvinyl chloride (PVC).

With respect to the “microcomputer”, since the original data does not distinguish between laptops and desktop computers, this article looks at them together for estimation. For the first estimation step, this article mainly refers to the data of Li (2015) for estimation [17], and for the second estimation step, this article mainly refers to the data of the Japanese magazine “Plastic Times”. The advantages of the estimation method in this paper are that firstly, it provides more accurate waste plastic content data for the “four machines and microcomputer”, which is a unique classification collection method in China; second, for the main types of waste plastics in China’s “four machines and microcomputer”, given the data on the proportion of waste plastics obtained through real dismantling, the results are more credible.

## 3. Results

### 3.1. Analysis of the Basic Situation of E-Waste Plastics in China

**Figure 1:**
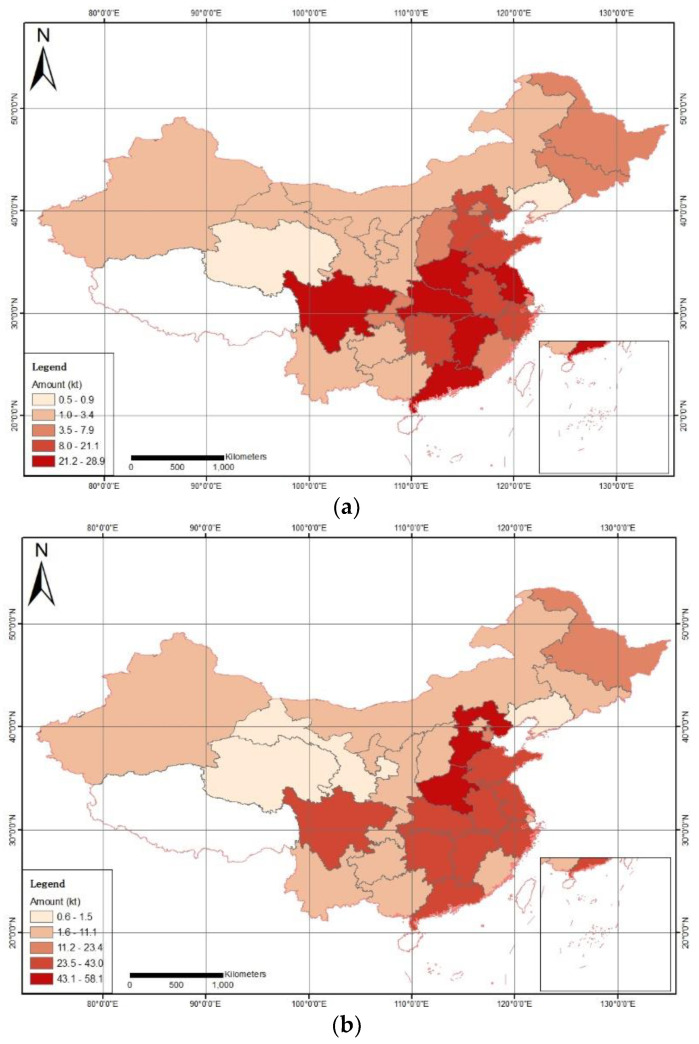
Generation amount of e-plastics in China: (**a**) in 2017; (**b**) average in 2012–2016.

According to static analysis, from the perspective of regions (see Table S1 of Supplementary Materials), the total annual output of e-plastics was ranked as East China, Central China, North China, Southwest China, South China, Northeast China, and Northwest China. In 2017, the total amount in East China ranking the first was 195,083 tons, and that in Northwest China which ranked the last was 20,473 tons. East China was 9.53 times of that in Northwest China. There were three regions with a total volume of more than 100,000 tons, namely 195,083 tons in East China, 121,337 tons in Central China, and 100,643 tons in North China (Figure 1a).

From the perspective of administrative regions, the top three with the most annual output of e-waste plastics were Henan Province (Central China), Hebei Province (North China) and Anhui Province (East China). The last three (in order from last to first) were Liaoning Province (Northeast China), Qinghai Province (Northwest China) and Gansu Province (Northwest China). In 2017, Henan Province ranked first with a total of 58,139 tons. Liaoning Province had a total of 569 tons, and Henan Province was more than 100 times that of Liaoning Province. There were 14 administrative regions with a total of more than 10,000 tons, of which there were five in East China (a total of seven), three in Central China (three in total), three in North China (five in total), and one in Southwest China (four in total), one in South China (two in total), one in the Northeast (three in total), none in the Northwest (five in total); two administrative regions with a total of below 1000 tons, one in the Northeast (three in total) and one in the Northwest (five in total).

According to dynamic analysis, in 2012, processing companies in the subsidy list of e-waste products processing funds (hereby referred to as processing companies) were set up in China. As of 2017, a total of 109 processing companies (none in Hainan and Tibet) were set up in 29 administrative regions in China. Six years later, the processing volume of processing companies was stabilized in a capacity level. In order to compare the development of various administrative regions, this article determines the average value of the total amount of e-waste plastics from 2012 to 2016 and compares it with the corresponding data in 2017.

From a regional perspective, the ranking order in terms of total annual e-waste plastics amount is follows: East China, Central China, North China, Southwest China, South China, Northeast China and Northwest China, and the ranking stays the same in terms of the average amount from 2012 to 2016 (SM Table S2). In 2017, the total amount of East China ranking the first was 195,083 tons, which was 1.59 times of the average from 2012 to 2016. The total amount of Northwest China ranking the last was 20,473 tons, which is 1.78 times of the average from 2012 to 2016. Both regions had a substantial increase. In 2017, the amount in East China was 9.53 times of that in Northwest China, and 10.65 times in terms of average from 2012 to 2016. In 2017, there were three regions with a total volume of more than 100,000 tons, namely 195,083 tons in East China, 121,337 tons in Central China, and 100,643 tons in North China. Only one region had a total volume of over 100,000 tons in terms of the average from 2012 to 2016, and that was East China with 122,601 tons.

From the perspective of administrative regions, the top three with the most annual output of e-waste plastics are the Henan Province (Central China), the Hebei Province (East North China), and the Anhui Province (East China). The last three (in order from back to front) are the Liaoning Province (Northeast Region), the Qinghai Province (Northwest Region) and the Gansu Province (Northwest Region). In terms of the average amount from 2012 to 2016, the Henan Province, among the top three, ranked from the third place to the first place. The Hebei and Anhui provinces replaced the Sichuan and Jiangsu provinces as the second and third places, respectively. Among the last three, the Gansu Province replaced the Inner Mongolia Autonomous Region as the third place. In 2017, the Henan Province ranked the first with a total volume of 58,139 tons, and the Liaoning Province ranked at the bottom with a total volume of 569 tons. The Henan Province is more than 100 times that in the Liaoning Province. In terms of the average from 2012 to 2016 (Figure 1b), the difference is nearly 60 times, which means a huge increase. The extreme difference in processing capacity has greatly expanded. In 2017, there were 14 administrative regions with a total of more than 10,000 tons, of which five were in East China (a total of seven), three were in Central China (three in total), three were in North China (five in total), and one was in Southwest China (four in total), one was in South China (two in total), one was in the Northeast (three in total), and none were in the Northwest (five in total). Compared to 2012 to 2016, two administrative regions, i.e., the Inner Mongolia Autonomous Region and the Heilongjiang Province, are new to the list. There are two administrative regions with a total volume of less than 1000 tons, including one in the Northeast Region (three in total) and one in the Northwest Region (five in total), which is the same as that during the period from 2012 to 2016.

### 3.2. Analysis on the Recovery Rate of China’s E-Waste Plastic Standards

It can be seen from Table 1 that excluding the initial year 2012, for a total of 5 years from 2013 to 2017, the highest standard recovery rate was 37.6% in 2015, and the lowest was 29.6%, a difference of eight percentage points. The 5-year average standard recovery rate was 33.3%. The standardized recycling rate was basically stable at one-third, which was lower than that of the agricultural sector (48%) and the transportation sector (42%), but higher than that of the construction sector (31%), the packaging sector (12%), and the daily-use sector (12%). There is still much room for improvement.

### 3.3. Typical Regional Model of E-Plastic Recycling in China

The abscissa is the relative abundance of renewable resources, which refers to the per capita e-waste plastics in each administrative region (tons/10,000 people)/the national per capita e-waste plastics (tons/10,000 people). The ordinate is the relative level of economic development, which refers to the per capita GDP of each administrative region (ten thousand yuan/person)/national per capita GDP (ten thousand yuan/person). The horizontal and vertical axes, respectively, take the national average value point as the origin, hence the four quadrants. Correspondingly, four types of areas are formed.

The characteristic of the first quadrant is that the relative abundance of renewable resources and the relative level of economic development is greater than the national average, indicating that the level of resource utilization is ascendant, and the level of economic development is high. The characteristic of the second quadrant is that the relative abundance of renewable resources is below the national level, and the relative level of economic development is greater than the national average, indicating that the level of resource utilization is low, but the level of economic growth is relatively high.

The characteristic of the third quadrant is that the relative abundance of renewable resources and the relative level of economic development are both lower than the national average, indicating that the level of resource utilization is inferior, while the level of economic development is low. The characteristics of the fourth quadrant are that the relative abundance of renewable resources is greater than the national level, and the relative level of economic development is less than the national average, indicating that the level of resource utilization is high, but the level of economic development remains low.

**Figure 2:**
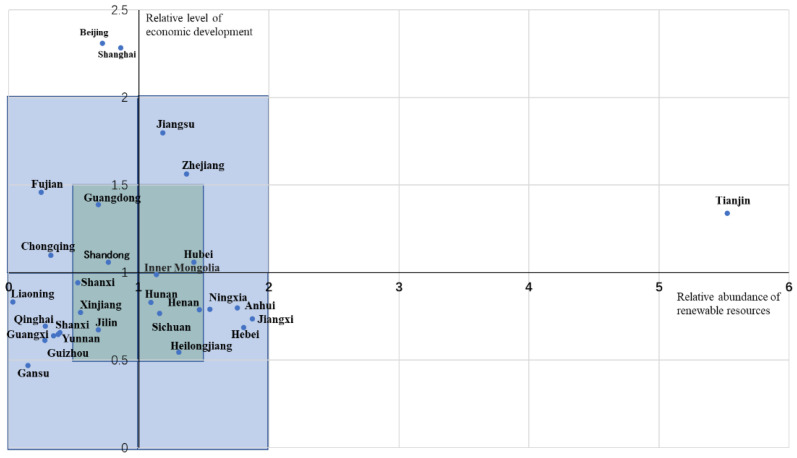
Provincial distribution of e-plastics recycling.

It can be seen from Figure 2 that the first quadrant includes four administrative regions of Tianjin, Hubei, Zhejiang and Jiangsu. The second quadrant includes six administrative regions of Shanghai, Shandong, Beijing, Guangdong, Chongqing and Fujian, and the third quadrant includes Jilin, Xinjiang, Shanxi, Shaanxi, Yunnan, Guizhou, Qinghai, Guangxi, Gansu, and Liaoning, as a total of ten administrative regions. The fourth quadrant covers Jiangxi, Hebei, Anhui, Ningxia, Henan, Heilongjiang, Sichuan, Inner Mongolia, and Hunan, as a total of nine administrative regions.

Each quadrant is further divided (Figure 2). With the origin as the center, rectangles with sides of 0.5 and one unit in each of the four quadrants are drawn to divide each quadrant into three regions to reflect the resources recycling and economic development level in the same quadrant. In Quadrant I, I = 0.5 includes one administrative region (Hubei), I = 1 includes two administrative regions (Zhejiang and Jiangsu), and I > 1 includes one administrative region (Tianjin). In Quadrant II, II = 0.5 includes two administrative regions (Shandong and Guangdong), II =1 includes two administrative regions (Chongqing and Fujian), and II> 1 includes two administrative regions (Shanghai and Beijing). In Quadrant III, III = 0.5 includes three administrative regions (Jilin, Xinjiang and Shaanxi), and III = 1 includes seven administrative regions (Shanxi Yunnan, Guizhou, Qinghai, Guangxi, Gansu and Liaoning). In IV quadrant, IV = 0.5 includes five administrative regions (Henan, Heilongjiang, Sichuan, Inner Mongolia and Hunan), IV = 1 includes four administrative regions (Jiangxi, Hebei, Anhui and Ningxia), and IV > 1 has no administrative regions.

**Figure 3:**
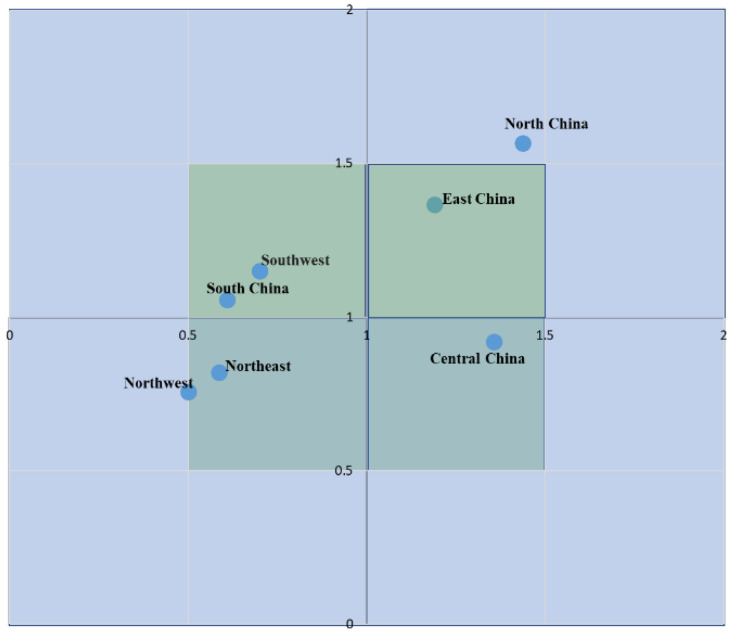
Regional distribution of e-plastics recycling.

Figure 3 shows the large-scale distribution of e-plastics in 2017. It can be seen from Figure 2 that the first quadrant includes two regions, i.e., North China and Northeast China, the second quadrant includes two regions, i.e., Southwest and South China, the third quadrant includes two regions, i.e., Northeast and Northwest, and the fourth quadrant includes 1 region, i.e., Central China. When each quadrant is further divided, as shown in Figure 3, in Quadrant I, I = 0.5 includes one region in East China, I = 1 includes one region in North China, and the rectangle with I > 1 has no region. In Quadrant II, the rectangle with I = 0.5 includes two regions, i.e., Southwest and South China, and II = 1 has no region, and II > 1 has no region. In Quadrant III, III = 0.5 includes two regions of Northeast and Northwest, and both III = 1 and III > 1 have no region. In Quadrant IV, IV = 0.5 includes one region of Central China, and both IV = 1 and IV > 1 have no region.

## 4. Discussion

The recycling of waste plastics in China’s electrical and electronic industries has dual attributes [20]. One is the improvement of resource utilization efficiency, and the other is the ecological and environmental protection effect brought by waste reduction.

**Figure 4:**
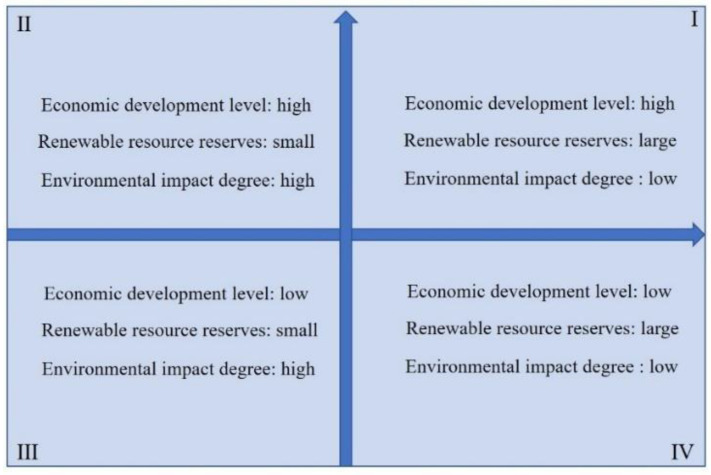
Classifying model of a typical model of e-plastics recycling.

Figure 4 is a conceptual model of a typical model of recycling e-waste plastics in China, based on the above two-dimensional coupling model [21]. Three typical patterns of resource utilization areas can be summarized as follows:

Model 1, resource-based demonstration area, quadrant 1, typical area, Tianjin
Mode 2, resource-based commissioned area, quadrant 2, typical area: Fujian
Model 3, resource-based traditional area, quadrant 3, typical area, Gansu
Model 4, resource-utilization potential area, quadrant 4, typical area, Heilongjiang

Model 1. This model is characterized by a high level of economic development, a large reserve of renewable resources, and a low degree of environmental impact. The typical area is Tianjin. The developed economy results in a large amount of household appliances per capita, and the spatial distribution density of renewable resources is relatively high. Thanks to a well-developed transportation system and a sound policy system, the operating cost of the collection and transportation system is lower. The saturated operation of standardized treatment facilities lowers the cost of resource utilization and improves efficiency. In turn, the recycled products obtained from resource utilization supplement the needs of economic development to a certain extent, and at the same time, the impact on the ecological environment in this process is correspondingly reduced.

Model 2. This model is characterized by a high level of economic development, a small reserve of renewable resources, and a high degree of environmental impact. The typical area is Fujian. The developed economy results in a large amount of household appliances per capita, and the spatial distribution density of renewable resources is relatively high. However, there are too few standardized treatment facilities (only one in 2017), which severely restricts the on-site treatment capacity. In turn, the recycled products obtained from resource utilization do not contribute much to the economic development. At the same time, the impact on the ecological environment has not been mitigated in this process.

Model 3. This model is characterized by a low level of economic development, a small reserve of renewable resources, and a high degree of environmental impact. The typical area is Gansu. The less developed economy results in small amount of per capita household appliances. The lower spatial distribution density of renewable resources, together with the less developed transportation system and imperfect policy system, leads to higher operation cost of the collection and transportation system. The unsaturated operation of standardized treatment facilities results in higher cost of resource utilization higher and lower efficiency. The recycled products obtained from resource utilization supplement the needs of economic development to a certain extent, thereby promoting economic development. At the same time, in this process, the impact on the ecological environment has decreased accordingly.

Model 4. This model is characterized by a low level of economic development, a large reserve of renewable resources, and a low degree of environmental impact. The typical area is Heilongjiang. A more developed transportation system and a sound policy system result in a lower operation cost of waste collection and transportation system. The saturated operation of standardized processing facilities leads to lower cost of resource utilization and higher efficiency. At the same time, in this process the impact on the ecological environment has decreased accordingly. Although the level of economic development is relatively low, high-efficiency resource utilization will facilitate local economy.

We found that the average recycling rate of e-plastics in China in the past five years was 33.1%, which reached the level of the European Union, the United States, and Japan [22,23]. The global recycling rate of all types of plastic was around 9% in 2015 [24,25]. While China’s recycling rate of plastic was much higher, there is still potential for further improvement room for e-plastic recycling.

## 5. Conclusions

On 4 August 2021, the Chinese government issued the “*Notice of the Three Departments on Encouraging Home Appliance Manufacturers to Carry out Recycling Target Responsibility Actions*”. This article identified four typical resource-based regional models and put forward differentiated suggestions. For the resource-based demonstration area, the priorities are to summarize the demonstration model, maintain the advantages of the demonstration, and expand the influence of the demonstration. It is also necessary to expand the standard dismantling targets, including small household appliances in the standard dismantling list during the “14th Five-Year Plan” period, and to explore an optimized path for collection and transportation. For resource-based entrusted areas, the priorities are to investigate the flow of cross-regional entrustment to find out the economic feasibility and potential environmental impact of cross-regional collection and transportation. There is also a need to explore the necessity of strengthening the implementation of standardized dismantling policies and improving on-site processing capabilities.

For traditional resource-based areas, the short-term priorities are to develop specific measures to optimize and standardize dismantling policies in the process of promoting economic transformation and development, and to reserve resource management space for further economic development. Moreover, it is necessary to explore the feasibility of developing a local resource recycling industry as a leading industry. Regarding the resource-based potential areas, the crucial tasks in the near term are: (i) to equip and standardize dismantling and other resource-based facilities catering to the economic development trends, (ii) to extend the resource-based industrial chain, and (iii) to explore the necessity and feasibility of setting up large-region resource-based dismantling centers and big data centers.

In summary, the differentiated recommendations are summarized largely as follows: maintain demonstration, strengthen policies, promote transformation, and tap potential. The focus of current work should be on resource-based demonstration areas and resource-based potential areas, and at the same time carrying out basic work for resource-based entrusted areas and resource-based traditional areas. In addition, it is also necessary to explore to build large-regional resource-based dismantling centers and big data centers in resource-based demonstration areas.

## Figures and Tables

**Table 1:** Standard recovery rate of waste plastics in four machines and microcomputer.

Year	2012	2013	2014	2015	2016	2017
Generation amount (kt) ^1^	748	914	1097	1295	1505	1723
Recycling amount (kt)	52	271	350	487	512	558
Recycling rate (%)	7	29.6	31.9	37.6	34	32.4

Note: ^1^ Data source from refs. [18,19].

## Data Availability

Not applicable.

## Supplementary Materials

The following are available online at https://www.mdpi.com/article/10.3390/ijerph19052807/s1, Table S1: The basic data of China’s e-plastics generation in 2017; Table S2: The basic data of China’ e-plastics generation from 2012 to 2016.
