# Supplementary material for: Examining the Temporal and Spatial Models of China’s Circular Economy Based upon Detailed Data of E-Plastic Recycling
Source: Int J Environ Res Public Health. 2022 Feb 28;19(5):2807. doi: 10.3390/ijerph19052807 (PMC8910096; doi:10.3390/ijerph19052807)
Supplement: Supplementary file 1 [file ijerph-19-02807-s001.zip › ijerph-1538188-supplementary.pdf]

Supplementary Materials

Examining the Temporal and Spatial Models of China's Circular Economy Based upon Detailed Data of E-plastics Recycling

Yu Qi [1,2], Ruying Gong [3], Xianlai Zeng [4,*], and Junfeng Wang [1,2,*]

* Correspondence: xlzeng@tsinghua.edu.cn (X. Zeng), Tel.: +86-10-6279-7163; jfwangnk@126.com (J. Wang)

Table S1 The basic data of China' e-plastics generation in 2017

Large area Sort	Large area Name	Administrative District Sort	Administrative District Name	Generation (tons)	Proportion in 7 regions (%)	Administrative District TOP 5	Administrative District Total sort
1	East China	1	Anhui	42964	0.08	3	3
		2	Jiangsu	40244	0.07	4	4
		3	Jiangxi	34157	0.06		6
		4	Zhejiang	34058	0.06		7
		5	Shandong	31003	0.06		10
		6	Shanghai	8575	0.02		15
		7	Fujian	4082	0.01		26
		Subtotal		195083	0.35		
2	Central China	1	Henan	58139	0.10	1	1
		2	Hubei	33939	0.06		8
		3	Hunan	29259	0.05		11
		Subtotal		121337	0.22		
3	North China	1	Hebei	54092	0.10	2	2
		2	Tianjin	23434	0.04		12
		3	Inner Mongolia	11144	0.02		14
		4	Beijing	6396	0.01		19
		5	Shanxi	5577	0.01		20
		Subtotal		100643	0.18		
4	Southwest	1	Sichuan	38826	0.07	5	5
		2	Yunnan	7215	0.01		17
		3	Guizhou	5304	0.01		23
		4	Chongqing	4125	0.01		25
		Subtotal		55470	0.10		
5	South China	1	Guangdong	33797	0.06		9
		2	Guangxi	5485	0.01		22
		Subtotal		39283	0.07		
6	Northeast	1	Heilongjiang	17948	0.03		13

		2	Jilin	7044	0.01		18
		3	Liaoning	569	0.00		29
		Subtotal		25561	0.05		
7	Northwest	1	Shaanxi	8361	0.01		16
		2	Xinjiang	5533	0.01		21
		3	Ningxia	4399	0.01		24
		4	Gansu	1511	0.00		27
		5	Qinghai	668	0.00		28
		Subtotal		20473	0.04		
Total				557849	1.00		

Table S2 The basic data of China' e-plastics generation from 2012 to 2016

Administrative District		Average yearly-generation amount (kt)	Proportion in 7 regions (%)	Administrative District TOP 5	Administrative District Total sort
Sort	Name				
5	Anhui	15.162	0.05	2	12
1	Jiangsu	28.566	0.09	5	2
2	Jiangxi	24.341	0.07		5
3	Zhejiang	21.097	0.06		7
3	Shandong	19.011	0.06		9
6	Shanghai	7.889	0.02		13
7	Fujian	6.534	0.02		16
Subtotal		122.601	0.37		
1	Henan	27.318	0.08	3	3
1	Hubei	27.097	0.08	4	4
3	Hunan	19.017	0.06		8
Subtotal		73.432	0.22		
1	Hebei	16.948	0.05		10
1	Tianjin	16.332	0.05		11
5	Inner Mongolia	2.003	0.01		27
3	Beijing	6.177	0.02		17
3	Shanxi	6.960	0.02		15
Subtotal		48.420	0.14		
1	Sichuan	28.913	0.09	1	1
3	Yunnan	2.401	0.01		24
3	Guizhou	2.093	0.01		25
2	Chongqing	5.313	0.02		19
Subtotal		38.720	0.12		
1	Guangdong	23.147	0.07		6

2	Guangxi	2.704	0.01	21
	Subtotal	25.851	0.08	
1	Heilongjiang	7.469	0.02	14
1	Jilin	5.743	0.02	18
3	Liaoning	0.487	0.00	29
	Subtotal	13.699	0.04	
1	Shaanxi	2.700	0.01	22
1	Xinjiang	3.350	0.01	20
1	Ningxia	2.089	0.01	26
1	Gansu	2.516	0.01	23
5	Qinghai	0.853	0.00	28
	Subtotal	11.507	0.03	
	Total	334.231	1.00	
